# Association between vitamin D levels and psychological stress-induced asthma prevalence: an untargeted metabolomics study combined with the NHANES database

**DOI:** 10.3389/fnut.2026.1784870

**Published:** 2026-04-22

**Authors:** Zhijie Zhang, Changren Bao, Haitian Li, Ruiheng Lan, Jingshu Luo, Yuchen Li, Kai Li, Xiaoyu Yang, Yuejia Wei, Siyang Yu, Jiafeng Shen, Mingsheng Lyu, Hongsheng Cui

**Affiliations:** 1 Beijing University of Chinese Medicine Third Affiliated Hospital, Beijing, China; 2 Beijing University of Chinese Medicine, Beijing, China

**Keywords:** asthma, NHANES database, psychological stress-induced asthma, untargeted metabolomics study, vitamin D

## Abstract

**Background:**

The incidence of psychological stress-induced asthma (PSA) is increasing annually, and numerous studies have shown that vitamin D is associated with the development of asthma, anxiety, and depression. However, to date, no studies have clarified the relationship between vitamin D levels and the prevalence of PSA. Therefore, this study aimed to integrate an analysis based on the National Health and Nutrition Examination Survey (NHANES) database with untargeted metabolomics to explore the potential association between vitamin D and PSA.

**Methods:**

This study integrates a cross-sectional analysis based on the NHANES database with a prospective cohort study incorporating untargeted metabolomics. A total of 2165 adults with asthma and 2331 adults with PSA from the NHANES cycles (2007–2012 and 2015–2018) were included to evaluate the association between vitamin D levels and the incidence of PSA. Multivariable logistic regression, restricted cubic spline modeling, and subgroup analyses were performed. For external validation, 60 adults with asthma and PSA were recruited, and untargeted metabolomics was used to measure plasma calcidiol levels. The association between vitamin D levels and PSA was assessed using regression models.

**Results:**

In the NHANES study, higher vitamin D levels were independently associated with a lower incidence of PSA (OR = 0.9967, *p* = 0.033). This association was particularly evident for depression-dominant PSA levels (OR = 0.9929, *p* < 0.001) and exhibited a significant non-linear relationship (*p*–non-linear = 0.04). When vitamin D levels were < 67 nmol/L, the incidence of PSA (depression) increased significantly (*p* = 0.02). Lower daily vitamin D intake was associated with significantly higher incidence rates of PSA and PSA (depression). In the Chinese cohort, higher calcidiol levels were potentially associated with a lower incidence of PSA (OR = 0.735, *p* = 0.0404), which warrants further validation in future studies. Sensitivity analyses confirmed the robustness of these findings with respect to PSA levels.

**Conclusion:**

Our findings suggest that lower vitamin D status may be associated with a higher likelihood of PSA. Specifically, lower circulating vitamin D levels and lower daily vitamin D intake were associated with higher odds of PSA. These observations may provide a potential basis for future research on the clinical evaluation and management of PSA levels.

## 1 Introduction

Asthma is a heterogeneous disease characterized by chronic airway inflammation and is primarily manifested by clinical symptoms, such as wheezing and dyspnea. Current guidelines recommend treatment regimens containing inhaled corticosteroids (ICS) as first-line therapy for asthma (1). Asthma is not a single inflammatory airway disease and is often accompanied by various comorbidities, including psychological disorders, such as depressive and anxiety disorders. Among these, anxiety and depression are the most common and have the broadest impact, and clinical studies have shown that anxiety and depression are closely associated with poor quality of life and inadequate symptom control in patients with asthma (1, 2). This asthma subtype, in which asthma symptoms are triggered or exacerbated in association with negative emotional states, is referred to as psychological stress-induced asthma (PSA). With further research into PSA, clinicians have gradually recognized that treating asthma comorbid with psychological disorders may contribute to asthma remission and a reduction in the use of corticosteroid medications (3, 4). However, the magnitude of these benefits remains insufficiently supported by robust evidence-based data (5).

Vitamin D is an essential nutrient for the human body that cannot be synthesized endogenously. Previous studies have shown that low vitamin D levels are associated with an increased risk of various common diseases; however, the specific causal relationships have not been fully established (6–8). A systematic review of vitamin D reported that reduced vitamin D levels across different age groups may be associated with an increased risk of respiratory tract infections. The review also found that children receiving vitamin D₃ supplementation had a significantly lower risk of asthma exacerbations than those who did not receive vitamin D₃ supplementation. These findings partially support an association between vitamin D and asthma exacerbations (9, 10). In addition to its association with asthma, vitamin D is also closely related to the development of anxiety and depression. The underlying mechanisms are primarily attributed to vitamin D acting as a neuroactive steroid regulator and a glucocorticoid antagonist that protects hippocampal function impaired by dysregulation of the HPA axis. However, whether vitamin D exerts a protective effect against anxiety and depression remains controversial in clinical studies (11–13). Meanwhile, it remains unclear whether vitamin D supplementation can reduce the occurrence of PSA.

With the increasing prevalence of asthma, growing attention has been directed toward psychiatric comorbidities such as anxiety and depression. However, the pathophysiological mechanisms underlying psychological stress-induced asthma remain poorly understood. Given its established roles in the development of both asthma and anxiety/depression, vitamin D deficiency may represent a potential pathogenic factor in the development of PSA. To address this knowledge gap, the present study combined data from the NHANES database with an untargeted metabolomics study to systematically investigate the association between vitamin D deficiency and the development of PSA.

## 2 Methods

### 2.1 Study design and population

#### 2.1.1 NHANES database selection

**Figure 1:**
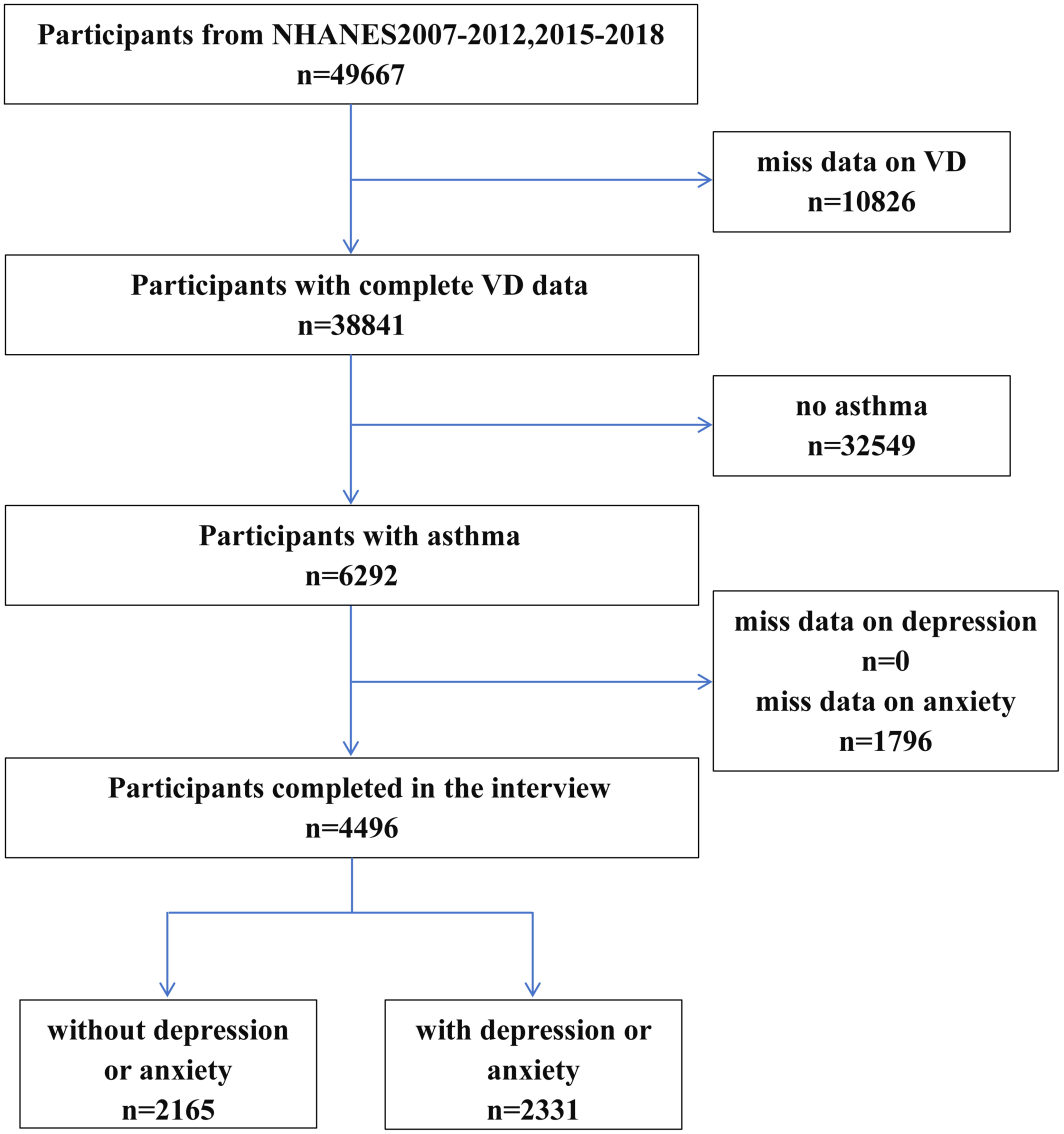
Flowchart of the participant selection.

The National Health and Nutrition Examination Survey (NHANES; https://wwwn.cdc.gov/nchs/nhanes/), conducted by the National Center for Health Statistics (NCHS) of the Centers for Disease Control and Prevention (CDC), is a nationally representative survey designed to assess the health and nutritional status of the civilian non-institutionalized population in the United States. The NHANES database is collected through household interviews and standardized physical examinations of survey participants. Each participant is assigned a sampling weight based on the proportion of the U.S. population that they represent. Data from the NHANES cycles (2007–2012 and 2015–2018) were included in this study. Of the initial 49,667 participants, those without serum vitamin D data (*n* = 10,826), without a diagnosis of asthma (*n* = 32,549), without a diagnosis of depression (*n* = 0), and without a diagnosis of anxiety (*n* = 1,796) were excluded, resulting in a final analytic sample of 4,496 participants. Among them, 2165 adults with asthma served as the control group, whereas 2331 adults with PSA were assigned to the PSA group (Figure 1). The NHANES protocol was approved by the NCHS Institutional Review Board, and written informed consent was obtained from all participants. No additional institutional approval was required for this study.

Definitions in the NHANES dataset were as follows: (1) Serum vitamin D concentrations were quantified using ultra-high-performance liquid chromatography–tandem mass spectrometry (UHPLC–MS/MS) to measure serum 25-hydroxyvitamin D₃ [25(OH)D₃] and 25-hydroxyvitamin D₂ [25(OH)D₂]. Total 25-hydroxyvitamin D [25(OH)D] was defined as the sum of 25(OH)D₂ and 25(OH)D₃. In this study, vitamin D refers to 25(OH)D. (2) Vitamin D intake was assessed using two 24-h dietary recall interviews. Dietary recalls were conducted using the Automated Multiple-Pass Method developed by the U.S. Department of Agriculture (14). During the first dietary recall, participants completed an in-person interview at a mobile examination center, reporting their dietary vitamin intake and supplement use. The second recall interview was conducted by telephone 3–10 days after the first interview to collect additional dietary information. Total vitamin D intake for each recall included both dietary sources and vitamin supplements reported by participants. The average daily vitamin D intake was calculated as the mean of total intake from the two consecutive recalls (15). (3) Asthma diagnosis was based on two primary criteria. The first criterion was derived from questionnaire data asking whether a doctor had ever told the participant that they had asthma (yes or no) (16). The second criterion involved pulmonary function testing, specifically bronchodilator responsiveness testing. A positive bronchodilator response was defined as an increase in forced expiratory volume in 1 s (FEV₁) of at least 12% from baseline or an absolute increase greater than 200 mL after bronchodilator administration (17). Participants meeting either of these criteria were classified as having asthma. (4) Depressive symptoms were assessed using the Patient Health Questionnaire-9 (PHQ-9). The PHQ-9 total score ranges from 0 to 27, with higher scores indicating more severe depressive symptoms (18). A PHQ-9 score ≥5 was considered indicative of possible depression. (5) Anxiety symptoms were assessed differently across NHANES cycles. For NHANES 2007–2012, anxiety was assessed by asking participants how many days in the past 30 days they felt worried, nervous, or anxious. Anxiety symptoms were categorized as “yes” (7–30 anxious days per month) or “no” (0–6 anxious days per month) (19). For NHANES 2015–2018, anxiety was assessed by asking how often participants felt worried, nervous, or anxious. Anxiety symptoms were classified as absent (less than once per month) or present (at least once per month) (20). (6) PSA was defined as asthma comorbid with either anxiety or depression. PSA (anxiety) referred to participants with asthma and anxiety without comorbid depression. PSA (depression) referred to participants with asthma and depression after excluding those with comorbid anxiety only. We were unable to determine whether asthma symptoms were directly triggered or exacerbated by psychological stress. Instead, PSA was operationalized based on the co-occurrence of asthma with anxiety and/or depression.

#### 2.1.2 External validation data selection

For external validation, a cohort of patients with PSA was recruited from the Beijing University of Chinese Medicine Third Affiliated Hospital between March 2025 and October 2025. The study protocol was approved by the Ethics Committee of the Beijing University of Chinese Medicine Third Affiliated Hospital (Approval No. BZYSY-2025YJSKTPJ-37). Written informed consent was obtained from all enrolled participants. The inclusion criteria for the PSA group were as follows: (1) Age ≥ 18 years. (2) Right-handedness. (3) Voluntary participation with good compliance and provision of written informed consent. (4) Asthma symptoms triggered or exacerbated by negative emotional states, with a Hamilton Anxiety Scale (HAMA) total score ≥7 or PHQ-9 total score ≥5. The inclusion criteria for the control (CON) group were as follows: (1) Sex- and age-matched participants enrolled during the same period who were able to complete all examinations and provided written informed consent. (2) Diagnosis of asthma according to the Guidelines for the Diagnosis and Management of Bronchial Asthma (2020 edition). (3) Asthma symptoms not triggered or exacerbated by negative emotional states, with a HAMA total score <7 and PHQ-9 total score <5. The exclusion criteria were as follows: (1) Presence of respiratory infectious diseases, chronic obstructive pulmonary disease (COPD), bronchiectasis, interstitial lung disease, allergic bronchopulmonary aspergillosis (ABPA), eosinophilia-associated lung diseases, or other respiratory disorders. (2) Comorbid conditions or medical histories that could affect brain function or related physiological systems, including heart failure, arrhythmia, transient ischemic attack, cerebrovascular disease, epilepsy, history of head trauma, chronic kidney disease, hematological disorders, malignancies, schizophrenia, dementia, or other psychiatric or cognitive disorders. (3) Pregnancy or lactation. (4) History of excessive alcohol consumption, defined as ethanol intake >80 g/day for men or >40 g/day for women for >5 years. (5) Current use of medications that may affect the sympathetic, parasympathetic, or central nervous system. (6) Inability to complete functional magnetic resonance imaging (fMRI) or presence of severe cranial anatomical abnormalities or lesions detected during fMRI. (7) Inability to complete pulmonary function testing, fractional exhaled nitric oxide (FeNO) measurement, blood sampling, or questionnaire assessments, including the Asthma Control Test (ACT). (8) Participation in other clinical drug trials within the previous 3 months. (9) Any other condition deemed by the investigators to make the participant unsuitable for study participation.

### 2.2 Exposure assessment

In the Chinese cohort, fasting venous blood samples were collected in the morning of the clinic visit using ethylenediaminetetraacetic acid (EDTA) anticoagulant tubes. After centrifugation at 3,000 rpm for 10 min at 4 °C, the supernatant plasma was collected and stored at −80 °C until analysis. After completion of participant enrollment, samples stored at −80 °C were removed and thawed on ice. An aliquot of 80 μL plasma was transferred into a 1.5 mL Eppendorf tube. Subsequently, 320 μL of methanol–acetonitrile protein precipitation solution (*v*/*v* = 2:1, containing mixed internal standards) was added, followed by vortexing for 1 min. Samples were ultrasonicated in an ice-water bath for 10 min and then incubated overnight at −40 °C. After centrifugation at 12,000 rpm for 20 min at 4 °C, 150 μL of the supernatant was transferred into LC–MS autosampler vials with inserts for analysis. Samples were analyzed using a liquid chromatography–mass spectrometry system (Waters ACQUITY UPLC I-Class Plus/Thermo Q Exactive HF). Chromatographic separation was performed using an ACQUITY UPLC HSS T3 column (100 mm × 2.1 mm, 1.8 μm). The column temperature was maintained at 45 °C. The mobile phases consisted of solvent A (water containing 0.1% formic acid) and solvent B (acetonitrile). The flow rate was set at 0.35 mL/min. The injection volume was 5 μL. The ion source was a heated electrospray ionization (HESI) source. Mass spectrometric data were acquired in both positive and negative ionization modes. Data acquisition was performed in data-dependent acquisition (DDA) mode using Full MS/dd-MS^2^ (Top 10). Raw data were processed using XCMS version 4.5.1 for baseline filtering, peak detection, integration, and retention time correction. Metabolite identification was based on retention time, accurate mass, MS/MS fragmentation patterns, and isotopic distribution. Identification was conducted by matching features against the Human Metabolome Database (HMDB), LipidMaps (v2.3), METLIN, and the local LuMet-Animal database. The quality assessment of the untargeted metabolomics data is presented in Supplementary Figure 1.

### 2.3 Outcome variables

In the NHANES study, the primary outcome variables were serum vitamin D levels and the occurrence of PSA. In the Chinese cohort, the outcome variables included calcifediol levels measured using untargeted metabolomics and the occurrence of PSA.

### 2.4 Covariates

A comprehensive set of covariates was selected to adequately account for potential confounding factors. The covariates in the NHANES study included age, sex, race/ethnicity, poverty-to-income ratio, marital status, hypertension, body mass index (BMI), physical activity level, white blood cell count (WBC#), lymphocyte percentage (LY%), segmented neutrophil percentage (NE%), and eosinophil percentage (EO%). The covariates in the Chinese cohort included age, sex, BMI, WBC#, LY%, NE%, and EO%.

### 2.5 Statistical analysis

In the Chinese cohort, compounds identified by untargeted metabolomics were screened based on qualitative identification scores, with a cutoff of 36 points (out of a maximum of 80), and compounds scoring below this threshold were considered inaccurately identified and excluded. After screening, data acquired in the positive and negative ionization modes were merged, and the combined data matrix was further processed as follows: (1) Relative standard deviation (RSD) filtering was applied by removing ion features with an RSD greater than 0.3 in quality control (QC) samples. (2) Missing value handling and zero-value imputation were performed by excluding ion features with more than 50% zero values within any group. The remaining zero values were imputed using half of the minimum ion intensity detected across all samples. After zero-value imputation, the data were log₂-transformed, and the resulting values were used as relative quantitative measures of metabolites (21–23).

In the NHANES analysis, five weighted logistic regression models were applied to evaluate the association between vitamin D levels and PSA. Model 1 was a univariable regression model examining the association between serum vitamin D levels and PSA. Model 2 was a univariable regression model that assessed the association between serum vitamin D levels and PSA (anxiety). Model 3 was a univariable regression model that evaluated the association between serum vitamin D levels and PSA (depression). Model 4 was adjusted for basic demographic variables, including sex, age, race/ethnicity, poverty-to-income ratio, and marital status. Model 5 was further adjusted for hypertension, body mass index (BMI), physical activity level, WBC#, LY%, NE%, and EO%. Restricted cubic spline (RCS) models were used to evaluate potential no*n*-linear relationships between serum vitamin D levels and PSA (depression). Subgroup analyses were conducted to examine the consistency of results across different stratifying variables, and the findings were visualized using forest plots. Sensitivity analyses were performed by excluding extreme values, followed by logistic regression analyses to assess the robustness of the results. In this study, age was categorized into quartiles (Q1–Q4) to evaluate the association between increasing age exposure levels and PSA occurrence. The lowest age quartile (Q1) served as the reference group, with Q2, Q3, and Q4 representing progressively higher age categories. A *p*-value for trend was calculated across quartiles to assess the dose–response relationship between age categories and PSA occurrence.

Descriptive statistics were used to summarize the baseline characteristics. The NHANES employed a complex multistage probability sampling design; therefore, sample weights and survey design variables were incorporated into all analyses to ensure nationally representative estimates for the U.S. population. Multiple imputation was applied to handle missing data in the relevant variables. *p*-values for the 39 differential metabolites were corrected for multiple testing using the Benjamini–Hochberg false discovery rate (FDR) procedure (24). Continuous variables were compared using the Student’s *t*-test, whereas categorical variables were compared using the chi-square test. Continuous variables were expressed as means ± standard error (SE) to describe central tendency and variability. Categorical variables were presented as unweighted counts (*n*) and weighted percentages (%). In the Chinese cohort, where sampling weights were not applied, continuous variables were expressed as means ± standard deviations (SD), and categorical variables were presented as frequencies (*n*) and percentages (%).

## 3 Results

### 3.1 Baseline characteristics of the study participants

The study population comprised participants from both the NHANES database and the Chinese cohort. In the NHANES database, a total of 4,496 participants from the (2007–2012 and 2015–2018) survey cycles were included, of whom 2165 reported asthma accompanied by anxiety or depression and 2331 reported asthma alone. Participants with asthma accompanied by anxiety or depression were classified into the PSA group, whereas those with asthma alone were classified into the control (CON) group. Compared to the CON group, participants with PSA were more prevalent among females, older individuals, those with lower income levels, and those with lower physical activity levels. Additionally, participants with PSA were more likely to have hypertension, lower EO%, and a higher WBC# (Table 1).

**Table 1:** All characteristics of the PSA and CON patients.

Variable	Demographic characteristics	CON	PSA	*p-*value
*N*		2,165	2,331	
Gender, %				<0.001
	Female	989 (48.1)	1,404 (61.4)	
	Male	1,176 (51.2)	927 (38.3)	
Age, years				<0.001
	0–25	755 (27.8)	526 (21.4)	
	26–40	409 (22.5)	540 (26.7)	
	41–56	359 (21.1)	582 (28.1)	
	57–90	642 (28.6)	683 (23.8)	
Race, %				0.127
	Mexican American	251 (6.0)	232 (5.6)	
	Non-Hispanic White	889 (68.9)	1,026 (67.3)	
	Non-Hispanic Black	584 (13.0)	545 (12.6)	
	Others	441 (12.1)	528 (14.5)	
PIR, %				<0.001
	0–1.29	622 (18.6)	949 (30.5)	
	1.3–3.49	752 (33.1)	738 (31.9)	
	3.5–5	791 (48.2)	644 (37.7)	
Marital, %				0.938
	Married or living with partners	930 (52.4)	1080 (52.2)	
	Living alone	1,235 (47.6)	1,251 (47.8)	
Hypertension, %				0.677
	Yes	911 (37.0)	999 (37.9)	
	No	1,254 (63.0)	1,332 (62.1)	
Hemameba, mmol/L		7.23 ± 2.34	7.65 ± 2.35	<0.001
Lymphocyte, mmol/L		30.09 ± 8.66	30.34 ± 8.48	0.497
Neutrophils, mmol/L		57.90 ± 9.86	58.29 ± 9.53	0.342
Eosinophils, mmol/L		3.38 ± 2.60	3.14 ± 2.42	0.038
BMI, Kg/m^2^		28.73 ± 7.31	30.43 ± 8.43	<0.001
Physical activity, %				<0.001
	Low	614 (26.4)	839 (32.4)	
	Middle	810 (39.9)	719 (31.9)	
	High	741 (33.9)	773 (35.7)	

A total of 60 patients were included in the Chinese cohort, comprising 29 patients in the PSA group and 31 patients in the CON group. In both groups, the proportion of female patients was higher than that of male patients. A significant difference in age was observed between the two groups (*p* = 0.014). When age was categorized into quartiles, a higher proportion of CON patients was observed in the younger age quartiles (Q1 and Q2), whereas a greater proportion of PSA patients was observed in the older age quartiles (Q3 and Q4). No significant differences were observed between the two groups in BMI, WBC#, LY%, NE%, or EO% (*p* > 0.05) (Table 2).

**Table 2:** Baseline characteristics of participants included in the Chinese cohort.

Variable	Total (*n* = 60)	CON (*n* = 31)	PSA (*n* = 29)	*p-*value
Gender (female, %)	40 (66.67)	21 (67.7)	19 (65.5)	1.000
Age (%)				0.014
Q1	12	9 (29)	3 (10.3)	
Q2	17	11 (35.5)	6 (20.7)	
Q3	18	9 (29)	9 (31)	
Q4	13	2 (6.5)	11 (37.9)	
BMI (Kg/m^2^, %)	23.71 (4.00)	23.68 (4.16)	23.74 (3.91)	0.954
WB [1,012/L, mean (SD)]	7.15 (2.03)	7.27 (1.6)	7.02 (2.43)	0.629
LY [%, mean (SD)]	28.94 (10.61)	29.61 (9.67)	28.21 (11.66)	0.614
NE [%, mean (SD)]	60.00 (12.03)	60.3 (10.13)	59.67 (13.95)	0.840
EO [%, mean (SD)]	3.71 (3.84)	4.29 (3.81)	3.09 (3.84)	0.227

### 3.2 Association between serum vitamin D levels and PSA incidence in the NHANES database

We first examined the association between vitamin D levels and PSA and further explored the associations of vitamin D levels with PSA (anxiety) and PSA (depression) using five regression models, as shown in Table 3. Model 1 demonstrated a statistically significant association between vitamin D levels and PSA (*p* = 0.0327). Model 2 showed no significant association between vitamin D levels and PSA (anxiety) (*p* = 0.5994). Model 3 revealed a strong association between vitamin D levels and PSA (depression) (*p* < 0.001). This association remained statistically significant after full adjustment for all covariates in Model 5 (*p* = 0.003). Vitamin D concentrations were subsequently categorized into deficient (≤50 nmol/L, Q1), insufficient (50 < VD < 75 nmol/L, Q2), sufficient (75 ≤ VD ≤ 125 nmol/L, Q3), and high (>125 nmol/L, Q4) levels and included as categorical variables in regression analyses (25). Using Q1 as the reference category, the OR and 95%CI for Q2, Q3, and Q4 in Model 5 were 0.7219 (0.5491–0.9492), 0.6725 (0.5333–0.8480), and 0.5901 (0.4215–0.8261), respectively. All models demonstrated statistically significant trends (*p* for trend < 0.05) (Table 3). Because the criteria used to define anxiety differed between the NHANES 2007–2012 and 2015–2018 cycles, we conducted cycle-specific analyses to evaluate the potential impact of this heterogeneity. Specifically, we performed univariable regression analyses within each cycle using Model 2 (univariable model, as defined in our study). We observed an association between serum vitamin D levels and PSA (anxiety) in the 2007–2012 cycles (*p* = 0.037), whereas no comparable association was detected in the 2015–2018 cycles (*p* = 0.599) (Supplementary Table 1).

**Table 3:** Association between VD and PSA in various models.

Variable	Model 1		Model 2		Model 3	
	OR (95%CI)	*p-*value	OR (95%CI)	*p-*value	OR (95%CI)	*p-*value
Continuous	0.9967 (0.9938–0.9997)	0.033	0.9993 (0.9965–1.0020)	0.599	0.9929 (0.9893–0.9964)	<0.001
VD (quartile)						
Q1	Ref		Ref		Ref	
Q2	0.7802 (0.6539–0.9309)	0.007	0.8759 (0.7249–1.0583)	0.167	0.6745 (0.5579–0.8155)	<0.001
Q3	0.7369 (0.5825–0.9322)	0.012	0.8650 (0.7042–1.0625)	0.164	0.5921 (0.4601–0.7619)	<0.001
Q4	0.7151 (0.4649–1.1000)	0.125	0.9405 (0.6148–1.4389)	0.775	0.5555 (0.3456–0.8928)	0.016
*p* for trend		0.015		0.309		<0.001

Variable	Model 4		Model 5	
	OR (95%CI)	*p-*value	OR (95%CI)	*p-*value
Continuous	0.9914 (0.9874–0.9955)	<0.001	0.9936 (0.9895–0.9977)	0.003
VD (quartile)				
Q1	Ref		Ref	
Q2	0.7324 (0.5975–0.8977)	0.003	0.7558 (0.6141–0.9302)	0.009
Q3	0.5965 (0.4555–0.7811)	<0.001	0.6568 (0.4960–0.8696)	0.004
Q4	0.5010 (0.2978–0.8428)	0.01	0.5890 (0.3446–1.0068)	0.053
*p* for trend		<0.001		0.005

**Figure 2:**
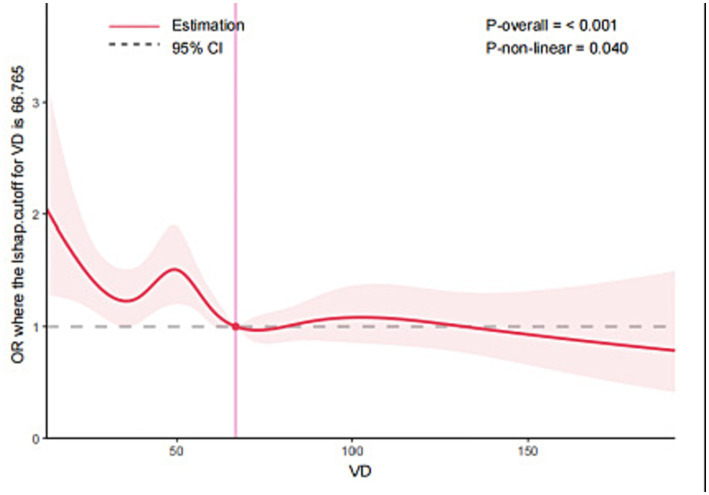
The RCS analysis of VD and asthma (depression).

In addition, RCS analysis demonstrated a negative non-linear association between vitamin D levels and PSA (depression) (*p*–non-linear = 0.040), with an inflection point at 67 nmol/L (Figure 2). To further investigate the threshold effect of vitamin D levels on PSA (depression), a two-piecewise linear regression model was applied. As shown in Table 4, a significant association was observed when vitamin D levels were < 67 nmol/L (*p* = 0.020). However, this association was no longer statistically significant when vitamin D levels exceeded this threshold (Table 4).

**Table 4:** Threshold effect analysis of VD and PSA (depression) by two-piecewise linear regression.

Variable	OR (95%CI)	*p-*value
<67 nmol/L	0.9881 (0.9784–0.9955)	0.020
≥67 nmol/L	1.0011 (0.9958–1.0064)	0.676

### 3.3 Subgroup analyses

**Figure 3:**
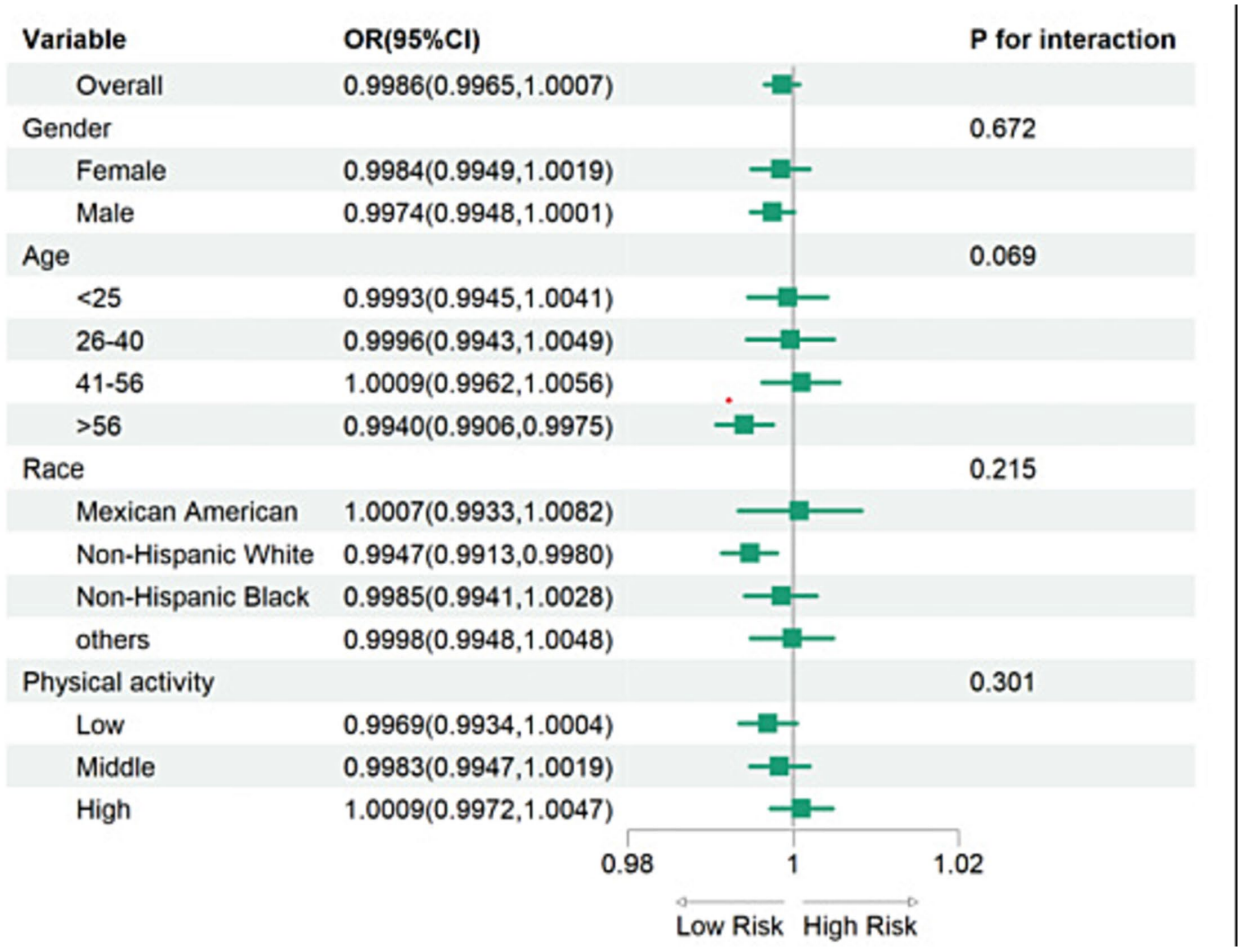
Forest plot of the relationship between VD and PSA (depression) in different subgroups.

Subgroup analyses were conducted to evaluate the consistency of the association between vitamin D levels and PSA (depression) across different population subgroups. ORs and 95% CIs for each subgroup were visualized using forest plots (Figure 3). The inverse association between vitamin D levels and PSA (depression) remained consistent across subgroups defined by sex, age, race/ethnicity, and physical activity. These findings indicate that the association between vitamin D levels and PSA (depression) was independent of these factors, with no significant interaction effects observed (all *p* for interaction > 0.05).

### 3.4 Association between vitamin D metabolites and PSA: Chinese cohort

#### 3.4.1 Vitamin D-related metabolites identified by untargeted metabolomics

**Figure 4:**
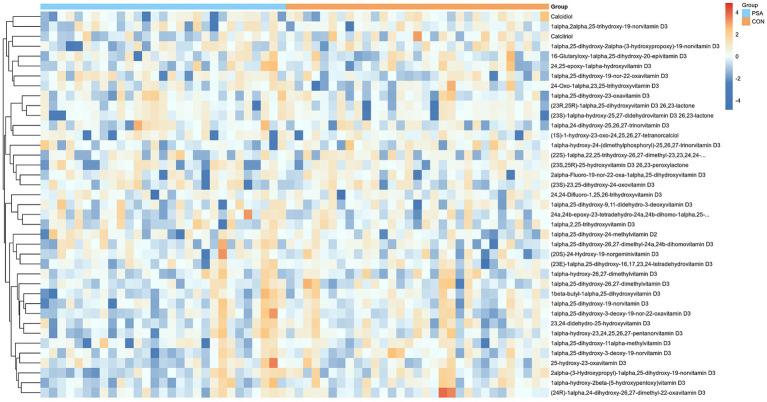
Heatmap of expression levels for 39 vitamin D metabolites.

Untargeted metabolomics was performed in 60 enrolled participants. Given the relatively small sample size, the metabolomic findings should be interpreted as exploratory. Significant differential metabolites were screened based on orthogonal partial least squares–discriminant analysis (OPLS-DA). A total of 39 vitamin D-related differential metabolites were identified. The expression patterns of these metabolites across samples are shown in Figure 4. Univariate analysis was conducted to evaluate the associations between all 39 vitamin D-related differential metabolites and the presence of PSA. The results showed that calcidiol was negatively associated with PSA incidence (*β* = −0.3076, *p* = 0.027; FDR = 0.72) (Supplementary Table 2). In addition, calcitriol exhibited a potential association with PSA, although this did not reach statistical significance (*β* = −0.2779, *p* = 0.067). The top 10 metabolites ranked by ascending *p*-values from the univariate analysis are presented in Table 5. The complete results are provided in Supplementary Tables 1, 2.

**Table 5:** Results of univariate analysis of variance for metabolites with significant differences in relationship to PSA onset (Top 10).

Variable	OR (95%CI)	*p-*value
Calcidiol	0.735 (0.564–0.959)	0.027
1alpha-hydroxy-2beta-(5-hydroxypentoxy)vitamin D3	0.821 (0.674–1)	0.054
Calcitriol	0.757 (0.565–1.015)	0.067
1alpha-hydroxy-24-(dimethylphosphoryl)-25,26,27-trinorvitamin D3	0.406 (0.143–1.151)	0.096
1alpha,25-dihydroxy-19-nor-22-oxavitamin D3	1.231 (0.966–1.568)	0.098
2alpha-(3-Hydroxypropyl)-1alpha,25-dihydroxy-19-norvitamin D3	0.869 (0.726–1.04)	0.133
1alpha,25-dihydroxy-2alpha-(3-hydroxypropoxy)-19-norvitamin D3	0.802 (0.601–1.072)	0.139
1alpha,25-dihydroxy-3-deoxy-19-norvitamin D3	0.851 (0.68–1.065)	0.164
1beta-butyl-1alpha,25-dihydroxyvitamin D3	0.886 (0.739–1.061)	0.194
24-Oxo-1alpha,23,25-trihydroxyvitamin D3	1.116 (0.943–1.32)	0.205

#### 3.4.2 Association between vitamin D and PSA incidence

In multivariable regression models adjusted for age, sex, BMI, WBC#, LY%, NE%, and EO%, calcidiol levels were consistently associated with PSA incidence and remained statistically significant (*p* = 0.0404). Additionally, when age was categorized into quartiles, the highest quartile (Q4) was significantly associated with PSA incidence (*p* = 0.015) (Table 6).

**Table 6:** Multivariate regression analysis results for the relationship between calcitriol and PSA incidence.

Variable	OR (95%CI)	*p-*value
(Intercept)	—	0.0215
Calcidiol	0.139 (0.022–0.878)	0.0404
Gender	0.756 (0.118–4.822)	0.7687
Age_q4Q2	0.664 (0.066–6.684)	0.7304
Age_q4Q3	3.906 (0.502–30.406)	0.2002
Age_q4Q4	28.755 (2.116–390.83)	0.015
WBC#	0.74 (0.451–1.214)	0.2407
LY%	0.641 (0.38–1.08)	0.1013
NE%	0.671 (0.402–1.119)	0.1325
EO%	0.636 (0.368–1.1)	0.1118
BMI	0.936 (0.746–1.173)	0.565

### 3.5 Association between daily vitamin D intake and PSA incidence

**Figure 5:**
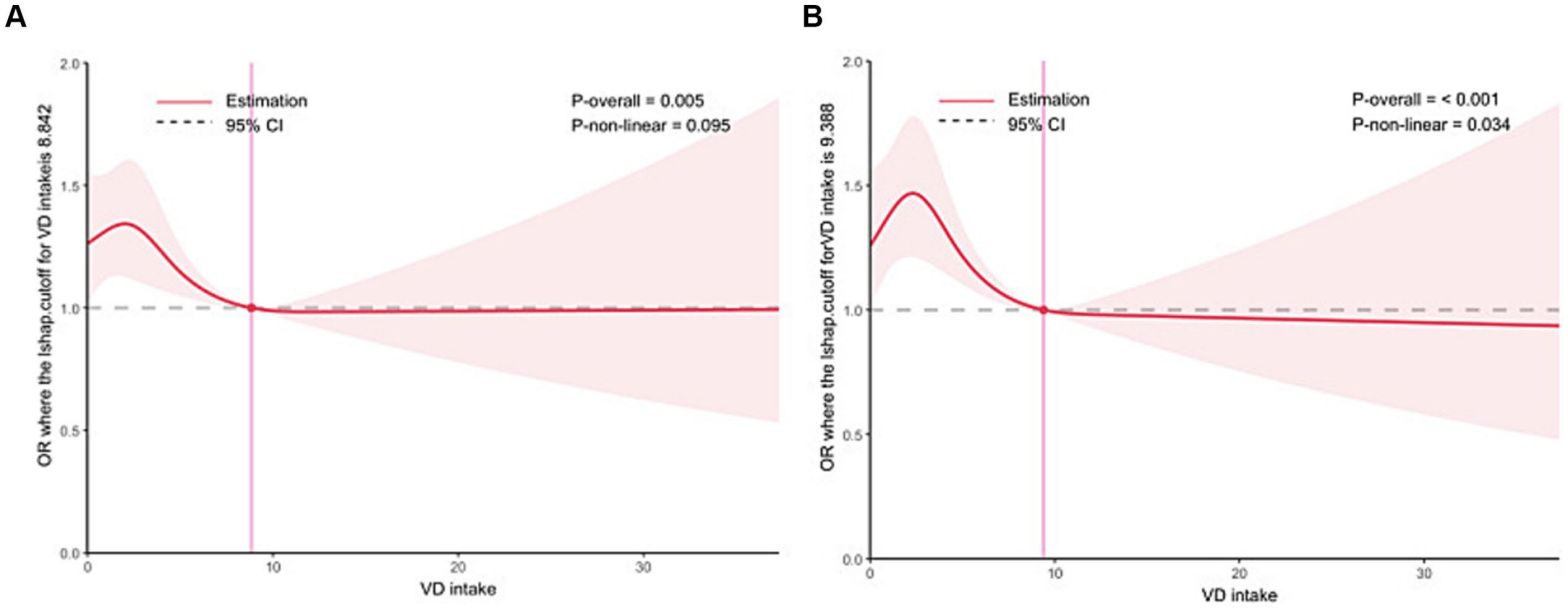
Relationship between vitamin D intake and PSA incidence. **(A)** Relationship between vitamin D intake and PSA, **(B)** relationship between vitamin D intake and PSA (depression).

To examine whether vitamin D intake is associated with the likelihood of PSA, we evaluated the relationship between daily vitamin D intake and PSA incidence. The results showed that daily vitamin D intake was inversely associated with both PSA (OR = 0.9793, *p* = 0.0190) and PSA (depression; OR = 0.9744, *p* = 0.0040). Furthermore, restricted cubic spline analysis indicated a significantly increased incidence of PSA when daily vitamin D intake was <9 μg/day. Similarly, the incidence of PSA (depression) increased significantly when daily vitamin D intake was <9 μg/day. These results are presented in Table 7 (Figure 5).

**Table 7:** The association between VD intake and PSA.

Variable	OR (95%CI)	*p-*value
VD intake—PSA	0.9793 (0.9626–0.9961)	0.0190
VD intake—PSA (depression)	0.9744 (0.9579–0.9912)	0.0040

### 3.6 Sensitivity analysis

A sensitivity analysis was conducted to assess the potential impact of extreme values on the study results. After excluding extreme vitamin D values (the highest and lowest 1%), the association between vitamin D levels and both PSA and PSA (depression) remained stable. When vitamin D levels were analyzed as a continuous variable, the fully adjusted model showed inverse associations with PSA (OR = 0.9951, *p* = 0.0207) and PSA (depression) (OR = 0.9950, *p* = 0.0404). When vitamin D levels were analyzed as categorical variables, the association between vitamin D levels and PSA (depression) remained unchanged (Table 8).

**Table 8:** Sensitivity analysis of VD, PSA, and PSA (depression).

Variable	PSA OR (95%CI)	*p-*value	PSA (depression) OR (95%CI)	*p-*value
Continuous	0.9951 (0.9910–0.9991)	0.0207	0.9950 (0.9902–0.9998)	0.0404
VD (quartile)				
Q1	Ref		Ref	
Q2	0.7691 (0.6256–0.9456)	0.0149	0.7578 (0.6158–0.9326)	0.0096
Q3	0.8187 (0.6659–1.0066)	0.0615	0.6592 (0.4961–0.8758)	0.0047
Q4	0.6917 (0.5219–0.9167)	0.0123	0.8133 (0.4262–1.5521)	0.5253
*p* for trend		0.0174		0.0236

## 4 Discussion

By analyzing data from both the NHANES database and the Chinese cohort, this study is the first to investigate the potential association between vitamin D deficiency and PSA. Similar patterns were observed across both datasets, suggesting that vitamin D deficiency may be associated with a higher likelihood of PSA. These findings are hypothesis-generating and may help guide future research into vitamin D-related strategies for the clinical evaluation and management of PSA; however, causal inferences and the pathogenesis of PSA remain unclear, and effective targeted therapies are currently lacking. Importantly, our untargeted clinical metabolomics analysis is the first to reveal differences in vitamin D and its related metabolites between patients with PSA and those with asthma alone, identifying calcidiol as a key metabolite. Over the past century, substantial advances have been made in vitamin D research, and its applications have gradually expanded to the prevention and management of asthma and other diseases (26). Ingested vitamin D is converted in the liver to 25-hydroxyvitamin D [25(OH)D], which is commonly used to reflect vitamin D status in populations because of its long half-life and relative stability against disease-related fluctuations (27, 28). Accordingly, serum 25(OH)D was selected in this study as the primary indicator of vitamin D status. In the NHANES study, PSA was associated with a higher WBC# and a lower EO%. These findings suggest that PSA may not be predominantly mediated by Th2-type cytokines. Consistently, untargeted metabolomic analysis in the Chinese cohort revealed associations between PSA incidence and both calcidiol and calcitriol levels, with calcidiol showing a particularly significant association (*p* = 0.027). These results provide external validation and complementary evidence for the strong association between vitamin D levels and PSA incidence observed in the NHANES study. Mechanistically, vitamin D may exert its effects by inhibiting CD4^+^ T cell proliferation and reducing the production of Th1 and IL-17-related cytokines. This is consistent with our previous experimental findings, which demonstrated a close association between PSA and IL-17-mediated pathways. Previous studies have also shown that vitamin D may enhance corticosteroid responsiveness and reduce steroid requirements in patients with IL-17 dominant asthma (29). However, the relationship between vitamin D and Th2-type cytokines remains unclear (30–33). Previous studies suggest that psychological stress-related asthma may be accompanied by alterations in neuroendocrine regulation. Under psychological stress, increases in inflammatory cells (e.g., monocytes and eosinophils), enhanced airway hyperresponsiveness, and dysregulated glucocorticoid receptor expression have been reported. As a nutrient with dual neuroendocrine and immunomodulatory properties, vitamin D may be relevant to immune regulation and inflammation in PSA, and its potential role warrants further investigation (34–37).

In the present study, we found that higher vitamin D intake may be associated with a lower likelihood of PSA. Previous clinical studies have demonstrated an association between vitamin D and acute asthma exacerbations. Vitamin D supplementation has been shown to reduce the frequency of asthma exacerbations and decrease corticosteroid use. These effects may be attributed to vitamin D-mediated enhancement of corticosteroid responsiveness (38, 39). Vitamin D also plays an important role in the treatment of anxiety and depression. Several clinical studies have reported that vitamin D supplementation is associated with improvements in anxiety and depressive symptoms in a dose-dependent manner. These therapeutic effects may be related to preservation of white matter integrity in the right inferior fronto-occipital fasciculus and enhanced functional connectivity between the right frontoparietal and medial visual networks. However, other studies have not demonstrated a significant benefit of vitamin D supplementation in alleviating anxiety or depressive symptoms. This inconsistency is consistent with our findings. Our findings suggest a threshold effect, whereby PSA risk increased markedly when daily vitamin D intake fell <9 ug/day, whereas higher intake levels did not confer additional significant risk reduction. This threshold effect may explain why some clinical trials report a protective effect of vitamin D supplementation, whereas others fail to demonstrate such benefits. Nevertheless, the underlying mechanisms remain unclear. At the same time, findings from other clinical studies that are inconsistent with ours should be interpreted objectively. Cross-study comparisons—particularly with respect to participant selection, population characteristics, and vitamin D intake assessment—may help explain heterogeneity and enable a more balanced interpretation of the overall evidence (40, 41). Increasing evidence suggests that vitamin D supplementation is particularly beneficial for individuals with low baseline serum 25(OH)D levels (42–46). Consistently, our results indicate a stronger association between serum vitamin D levels and PSA risk when vitamin D concentrations are <67 nmol/L. Collectively, these findings suggest that among asthma patients with low vitamin D levels or insufficient dietary vitamin D intake, monitoring vitamin D status should be prioritized to effectively mitigate the risk of PSA. These results provide additional evidence to support existing consensus statements and clinical guidelines (47).

A major strength of this study is its integrative design, which combines an NHANES-based cross-sectional analysis with a clinical prospective cohort incorporating untargeted metabolomics. This framework allowed us to examine potential associations between vitamin D (and related metabolites) and PSA, providing complementary evidence beyond the NHANES findings. Moreover, we observed that higher vitamin D intake was associated with a lower likelihood of PSA; however, these results should be interpreted cautiously, as they do not establish causality. Nevertheless, several limitations should be acknowledged. In the NHANES study, first, the retrospective assessment of vitamin D intake was subject to incomplete data. Second, anxiety was not diagnosed using standardized anxiety assessment scales. Third, PSA in this study represents an operational construct rather than an established diagnostic entity, which may introduce phenotype misclassification (e.g., asthma with psychiatric comorbidity vs. true stress-related asthma) and potentially bias the estimated associations. In the clinical cohort study, first, the relatively small sample size limited further investigation into the associations between vitamin D and its metabolites and asthma comorbid with anxiety or depression separately. Second, although untargeted metabolomics was applied to comprehensively characterize vitamin D metabolism, absolute quantification of individual vitamin D metabolites was not performed. Third, as the clinical study is an ongoing prospective cohort, subsequent follow-up outcomes could not yet be incorporated into the present analysis. Future studies with extended follow-up of the clinical cohort are warranted to further confirm the association between vitamin D deficiency and the development of PSA.

## 5 Conclusion

Overall, our findings suggest that vitamin D deficiency may be associated with an increased risk of psychological stress-induced asthma (PSA). Specifically, lower serum vitamin D levels were associated with higher odds of PSA, and lower daily vitamin D intake showed a similar association with increased odds of PSA. These observations may help inform future research on vitamin D-related indicators for the clinical evaluation of PSA.

## Data Availability

The data presented in this study are deposited in the MetaboLights repository, accession number MTBLS14208 (https://www.ebi.ac.uk/metabolights/MTBLS14208). NHANES data are publicly available at https://www.cdc.gov/nchs/nhanes/.

## References

[1] Global Initiative for Asthma. Global strategy for asthma management and prevention, (2025). Available online at: http://www.ginasthma.org (Accessed 15 November 2025).

[2] Boulet LP. Influence of comorbid conditions on asthma. Eur Respir J. (2009) 33:897–906. doi: 10.1183/09031936.00121308, 19336592

[3] Yorke J Fleming SL Shuldham CM. Psychological interventions for adults with asthma. Cochrane Database Syst Rev. (2006) 2006:CD002982 doi: 10.1002/14651858.CD002982.pub3 16437449 PMC7004249

[4] Parry GD Cooper CL Moore JM Yadegarfar G Campbell MJ Esmonde L . Cognitive behavioural intervention for adults with anxiety complications of asthma: prospective randomised trial. Respir Med. (2012) 106:802–10. doi: 10.1016/j.rmed.2012.02.006, 22398158

[5] Agarwal CD Palka JM Gajewski AJ Khan DA Brown ES. The efficacy of citalopram or escitalopram in patients with asthma and major depressive disorder. Ann Allergy Asthma Immunol. (2023) 132:374–82. doi: 10.1016/j.anai.2023.11.004 37952772

[6] Bouillon R Manousaki D Rosen C Trajanoska K Rivadeneira F Richards JB. The health effects of vitamin D supplementation: evidence from human studies. Nat Rev Endocrinol. (2021) 18:96–110. doi: 10.1038/s41574-021-00593-z, 34815552 PMC8609267

[7] Harrison SR Li D Jeffery LE Raza K Hewison M. Vitamin D, autoimmune disease and rheumatoid arthritis. Calcif Tissue Int. (2019) 106:58–75. doi: 10.1007/s00223-019-00577-2, 31286174 PMC6960236

[8] Latic N Erben RG. Vitamin D and cardiovascular disease, with emphasis on hypertension, atherosclerosis, and heart failure. Int J Mol Sci. (2020) 21:6483. doi: 10.3390/ijms21186483, 32899880 PMC7555466

[9] Shah VP Nayfeh T Alsawaf Y Saadi S Farah M Zhu Y . A systematic review supporting the Endocrine Society clinical practice guidelines on vitamin D. J Clin Endocrinol Metab. (2024) 109:1961–74. doi: 10.1210/clinem/dgae312, 38828942

[10] Urashima M Segawa T Okazaki M Kurihara M Wada Y Ida H. Randomized trial of vitamin D supplementation to prevent seasonal influenza a in schoolchildren. Am J Clin Nutr. (2010) 91:1255–60. doi: 10.3945/ajcn.2009.29094, 20219962

[11] Obradovic D Gronemeyer H Lutz B Rein T. Cross-talk of vitamin D and glucocorticoids in hippocampal cells. J Neurochem. (2005) 96:500–9. doi: 10.1111/j.1471-4159.2005.03579.x, 16336217

[12] Eyles DW Smith S Kinobe R Hewison M McGrath JJ. Distribution of the vitamin D receptor and 1 alpha-hydroxylase in human brain. J Chem Neuroanat. (2005) 29:21–30. doi: 10.1016/j.jchemneu.2004.08.006, 15589699

[13] Mikola T Marx W Lane MM Hockey M Loughman A Rajapolvi S . The effect of vitamin D supplementation on depressive symptoms in adults: a systematic review and meta-analysis of randomized controlled trials. Crit Rev Food Sci Nutr. (2022) 63:11784–801. doi: 10.1080/10408398.2022.2096560, 35816192

[14] Xie J Tian Z Liu X Yu X Hu X Liu K. Association of C-reactive protein-albumin-lymphocyte (CALLY) index with all-cause mortality in patients with type 2 diabetes: results from the retrospective cohort study of NHANES 1999-2010. Medicine (Baltimore). (2025) 104:e45634. doi: 10.1097/MD.0000000000045634, 41204477 PMC12599718

[15] Lai Z Jin F Zhu C Zhu Z. No independent association between dietary calcium/vitamin D and appendicular lean mass index in middle-aged women: NHANES cross-sectional analysis (2011–2018). Sci Rep. (2025) 15:17290. doi: 10.1038/s41598-025-02505-x, 40389590 PMC12089267

[16] Kohandel Gargari O Fathi M Rajai Firouzabadi S Mohammadi I Mahmoudi MH Sarmadi M . Assessing the diagnostic accuracy of machine learning algorithms for identification of asthma in United States adults based on NHANES dataset. Sci Rep. (2025) 15:4537. doi: 10.1038/s41598-025-88345-1, 39915528 PMC11802912

[17] Trepić N Nemet M Vukoja M. Assessing the impact of the updated 2021 European Respiratory Society/American Thoracic Society criteria on bronchodilator responsiveness in asthma. Cureus. (2024) 16:e66844 doi: 10.7759/cureus.66844 39280484 PMC11395171

[18] Kroenke K Spitzer RL Williams JB. The PHQ-9: validity of a brief depression severity measure. J Gen Intern Med. (2001) 16:606–13. doi: 10.1046/j.1525-1497.2001.016009606.x, 11556941 PMC1495268

[19] Wen Z Bai L Wu S Chen J Jama HA Sawmadal JD. Association of serum vitamin D with anxiety in US adults: a cross-sectional study. Front Nutr. (2024) 11:1371170. doi: 10.3389/fnut.2024.1371170, 38549749 PMC10973008

[20] Fardell JE Irwin CM Vardy JL Bell ML. Anxiety, depression, and concentration in cancer survivors: National Health and nutrition examination survey results. Support Care Cancer. (2023) 31:272. doi: 10.1007/s00520-023-07710-w, 37060376 PMC10105664

[21] Paul I Bolzan D Youssef A Gagnon KA Hook H Karemore G . Parallelized multidimensional analytic framework applied to mammary epithelial cells uncovers regulatory principles in EMT. Nat Commun. (2023) 14:688. doi: 10.1038/s41467-023-36122-x, 36755019 PMC9908882

[22] Hyötyläinen T McGlinchey A Salihovic S Schubert A Douglas A Hay DC . In utero exposures to perfluoroalkyl substances and the human fetal liver metabolome in Scotland: a cross-sectional study. Lancet Planet Health. (2024) 8:e5–e17. doi: 10.1016/s2542-5196(23)00257-7, 38199723

[23] Colas S Marie B Morin S Milhe-Poutingon M Foucault P Chalvin S . New sensitive tools to characterize meta-metabolome response to short- and long-term cobalt exposure in dynamic river biofilm communities. Sci Total Environ. (2024) 927:171851. doi: 10.1016/j.scitotenv.2024.171851, 38518822

[24] Victor A Elsässer A Hommel G Blettner M. Judging a plethora of p-values: how to contend with the problem of multiple testing--part 10 of a series on evaluation of scientific publications. Dtsch Arztebl Int. (2010) 107:50–6. doi: 10.3238/arztebl.2010.0050, 20165700 PMC2822959

[25] Płudowski P Kos-Kudła B Walczak M Fal A Zozulińska-Ziółkiewicz D Sieroszewski P . Guidelines for preventing and treating vitamin D deficiency: a 2023 update in Poland. Nutrients. (2023) 15:695. doi: 10.3390/nu15030695, 36771403 PMC9920487

[26] Gallagher JC Rosen CJ. Vitamin D: 100 years of discoveries, yet controversy continues. Lancet Diabetes Endocrinol. (2023) 11:362–74. doi: 10.1016/s2213-8587(23)00060-8 37004709

[27] Paul G Brehm JM Alcorn JF Holguín F Aujla SJ Celedón JC. Vitamin D and asthma. Am J Respir Crit Care Med. (2011) 185:124–32. doi: 10.1164/rccm.201108-1502CI 22016447 PMC3297088

[28] Hollis BW. Assessment and interpretation of circulating 25-hydroxyvitamin D and 1,25-dihydroxyvitamin D in the clinical environment. Rheum Dis Clin N Am. (2012) 38:29–44. doi: 10.1016/j.rdc.2012.03.005, 22525841

[29] Pfeffer PE Hawrylowicz CM. Vitamin D in asthma: mechanisms of action and considerations for clinical trials. Chest. (2017) 153:1229–39. doi: 10.1016/j.chest.2017.09.005, 28923762

[30] Adorini L Penna G Giarratana N Roncari A Amuchastegui S Daniel KC . Dendritic cells as key targets for immunomodulation by vitamin D receptor ligands. J Steroid Biochem Mol Biol. (2004) 89-90:437–41. doi: 10.1016/j.jsbmb.2004.03.013 15225816

[31] Mahon BD Wittke A Weaver V Cantorna MT. The targets of vitamin D depend on the differentiation and activation status of CD4 positive T cells. J Cell Biochem. (2003) 89:922–32. doi: 10.1002/jcb.10580, 12874827

[32] Tang J Zhou R Luger D Zhu W Silver PB Grajewski RS . Calcitriol suppresses antiretinal autoimmunity through inhibitory effects on the Th17 effector response. J Immunol. (2009) 182:4624–32. doi: 10.4049/jimmunol.0801543, 19342637 PMC2756755

[33] Lange NE Litonjua A Hawrylowicz CM Weiss S. Vitamin D, the immune system and asthma. Expert Rev Clin Immunol. (2009) 5:693–702. doi: 10.1586/eci.09.53, 20161622 PMC2812815

[34] Ohno I. Neuropsychiatry phenotype in asthma: psychological stress-induced alterations of the neuroendocrine-immune system in allergic airway inflammation. Allergol Int. (2017) 66S:S2–8. doi: 10.1016/j.alit.2017.06.005, 28669635

[35] Di Rosa M Malaguarnera M Nicoletti F Malaguarnera L. Vitamin D3: a helpful immuno-modulator. Immunology. (2011) 134:123–39. doi: 10.1111/j.1365-2567.2011.03482.x, 21896008 PMC3194221

[36] Di Rosa M Malaguarnera G De Gregorio C Palumbo M Nunnari G Malaguarnera L. Immuno-modulatory effects of vitamin D3 in human monocyte and macrophages. Cell Immunol. (2012) 280:36–43. doi: 10.1016/j.cellimm.2012.10.009 23261827

[37] Valle MS Russo C Casabona A Crimi N Crimi C Colaianni V . Anti-inflammatory role of vitamin D in muscle dysfunctions of patients with chronic obstructive pulmonary disease: a comprehensive review. Minerva Med. (2022) 114:357–71. doi: 10.23736/S0026-4806.22.07879-X 35332756

[38] Jolliffe DA Greenberg L Hooper RL Griffiths CJ Camargo CA Kerley CP . Vitamin D supplementation to prevent asthma exacerbations: a systematic review and meta-analysis of individual participant data. Lancet Respir Med. (2017) 5:881–90. doi: 10.1016/s2213-2600(17)30306-5, 28986128 PMC5693329

[39] Xystrakis E Kusumakar S Boswell S Peek E Urry Z Richards DF . Reversing the defective induction of IL-10-secreting regulatory T cells in glucocorticoid-resistant asthma patients. J Clin Invest. (2005) 116:146–55. doi: 10.1172/JCI21759, 16341266 PMC1307558

[40] Okereke OI Reynolds CF Mischoulon D Chang G Vyas CM Cook NR . Effect of long-term vitamin D3 supplementation vs placebo on risk of depression or clinically relevant depressive symptoms and on change in mood scores: a randomized clinical trial. JAMA. (2020) 324:471–80. doi: 10.1001/jama.2020.10224 32749491 PMC7403921

[41] Jorde R Sneve M Figenschau Y Svartberg J Waterloo K. Effects of vitamin D supplementation on symptoms of depression in overweight and obese subjects: randomized double blind trial. J Intern Med. (2008) 264:599–609. doi: 10.1111/j.1365-2796.2008.02008.x, 18793245

[42] Zhao W Zhu D-M Shen Y Zhang Y Chen T Cai H . The protective effect of vitamin D supplementation as adjunctive therapy to antidepressants on brain structural and functional connectivity of patients with major depressive disorder: a randomized controlled trial. Psychol Med. (2024) 54:2403–13. doi: 10.1017/S0033291724000539, 38482853

[43] de Koning EJ Lips P Penninx BWJH Elders PJM Heijboer AC den Heijer M . Vitamin D supplementation for the prevention of depression and poor physical function in older persons: the D-Vitaal study, a randomized clinical trial. Am J Clin Nutr. (2019) 110:1119–30. doi: 10.1093/ajcn/nqz141, 31340012 PMC6821546

[44] Dawson-Hughes B. Vitamin D and muscle function. J Steroid Biochem Mol Biol. (2017) 173:313–6. doi: 10.1016/j.jsbmb.2017.03.018, 28341251

[45] Parker GB Brotchie H Graham RK. Vitamin D and depression. J Affect Disord. (2016) 208:56–61. doi: 10.1016/j.jad.2016.08.082 27750060

[46] Reid IR. High-dose vitamin D: without benefit but not without risk. J Intern Med. (2018) 284:694–6. doi: 10.1111/joim.12836 30230641

[47] Demay MB Pittas AG Bikle DD Diab DL Kiely ME Lazaretti-Castro M . Vitamin D for the prevention of disease: an Endocrine Society clinical practice guideline. J Clin Endocrinol Metab. (2024) 109:1907–47. doi: 10.1210/clinem/dgae290, 38828931

