# Supplementary material for: Association between vitamin D levels and psychological stress-induced asthma prevalence: an untargeted metabolomics study combined with the NHANES database
Source: Front Nutr. 2026 Apr 22;13:1784870. doi: 10.3389/fnut.2026.1784870 (PMC13144065; doi:10.3389/fnut.2026.1784870)
Supplement: Supplementary file 1 [file Supplementary_file_1.DOCX]

Supplementary Material

# 1 Supplementary Figure 1

#
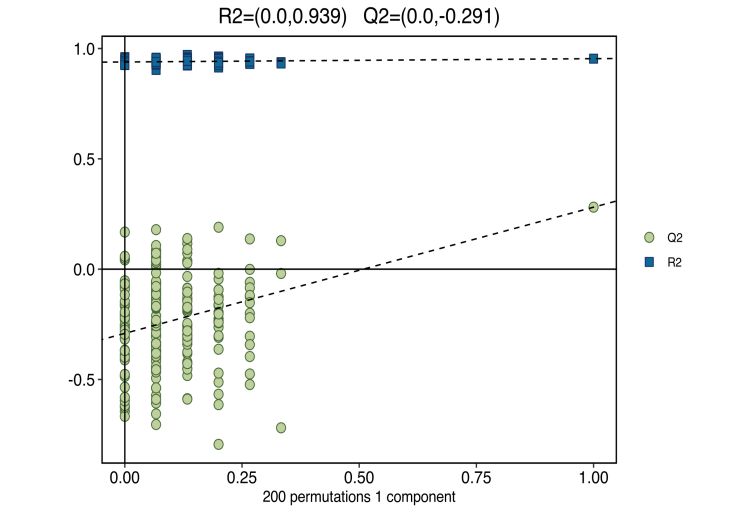

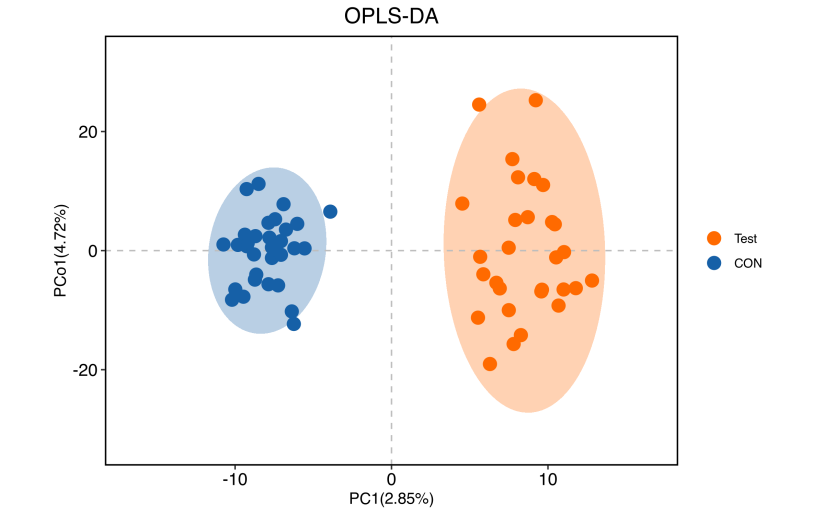


1. （B）

Figure 1 Metabolomics OPLS-DA Analysis and Permutation Analysis,(A)OPLS-DA plot,(B)Permutation plot

# 2 Supplementary Table 1

Table 2 The Association Between VD and PSA（anxiety） in Model 2 (2007-2012,2015-2018)

|  | **Model 2(2007-2012)** | | **Model 2(2015-2018)** | |
| --- | --- | --- | --- | --- |
|  | **OR(95%CI)** | **P value** | **OR(95%CI)** | **P value** |
| Continuous | 0.9956(0.9916,0.9996) | 0.037 | 0.9990(0.9952,1.0027) | 0.599 |
| VD(quartile) |  |  |  |  |
| Q1 | 0.9956 (0.9915-0.9997) | 0.0367 | 0.999 (0.9951-1.0029) | 0.5989 |
| Q2 | 0.667 (0.5225-0.8513) | 0.0017 | 1.0427 (0.7005-1.5521) | 0.831 |
| Q3 | 0.8688 (0.6668-1.1319) | 0.29 | 1.1302 (0.718-1.779) | 0.5843 |
| Q4 | 0.6119 (0.4529-0.8266) | 0.0019 | 0.9478 (0.6707-1.3394) | 0.7527 |

# 3 Supplementary Table 2

Table 2 Results of Univariate Analysis of Variance for Metabolites with Significant Differences in Relationship to PSA Onset

| **Rank** | **Metabolite Name** | **β** | **SE** | **OR 95%（CI）** | ***P* value** | **FDR** |
| --- | --- | --- | --- | --- | --- | --- |
| 1 | Calcidiol | -0.30757 | 0.13576 | 0.735(0.563-0.959) | 0.0272 | 0.72 |
| 2 | 1alpha-hydroxy-2beta-(5-hydroxypentoxy)vitamin D3 | -0.1974 | 0.10053 | 0.821(0.674-1) | 0.0544 | — |
| 3 | Calcitriol | -0.27786 | 0.14911 | 0.757(0.565-1.014) | 0.0675 | — |
| 4 | 1alpha-hydroxy-24-(dimethylphosphoryl)-25,26,27-trinorvitamin D3 | -0.90294 | 0.53302 | 0.405(0.143-1.152) | 0.0956 | — |
| 5 | 1alpha,25-dihydroxy-19-nor-22-oxavitamin D3 | 0.20776 | 0.12362 | 1.231(0.966-1.568) | 0.0982 | — |
| 6 | 2alpha-(3-Hydroxypropyl)-1alpha,25-dihydroxy-19-norvitamin D3 | -0.14038 | 0.09205 | 0.869(0.726-1.041) | 0.1327 | — |
| 7 | 1alpha,25-dihydroxy-2alpha-(3-hydroxypropoxy)-19-norvitamin D3 | -0.22019 | 0.14692 | 0.802(0.602-1.07) | 0.1394 | — |
| 8 | 1alpha,25-dihydroxy-3-deoxy-19-norvitamin D3 | -0.16104 | 0.11412 | 0.851(0.681-1.065) | 0.1635 | — |
| 9 | 1beta-butyl-1alpha,25-dihydroxyvitamin D3 | -0.12145 | 0.09242 | 0.886(0.739-1.062) | 0.194 | — |
| 10 | 24-Oxo-1alpha,23,25-trihydroxyvitamin D3 | 0.10931 | 0.08535 | 1.116(0.944-1.319) | 0.2054 | — |
| 11 | 1alpha,25-dihydroxy-24-methylvitamin D2 | 0.09122 | 0.07406 | 1.096(0.947-1.267) | 0.223 | — |
| 12 | 1alpha,24-dihydroxy-25,26,27-trinorvitamin D3 | 0.05416 | 0.04581 | 1.056(0.965-1.155) | 0.2419 | — |
| 13 | 24a,24b-epoxy-23-tetradehydro-24a,24b-dihomo-1alpha,25-dihydroxyvitamin D3 | 0.15875 | 0.13852 | 1.172(0.893-1.538) | 0.2565 | — |
| 14 | (24R)-1alpha,24-dihydroxy-26,27-dimethyl-22-oxavitamin D3 | -0.1171 | 0.1025 | 0.889(0.728-1.087) | 0.2579 | — |
| 15 | (20S)-24-Hydroxy-19-norgeminivitamin D3 | 0.0776 | 0.07092 | 1.081(0.94-1.242) | 0.2784 | — |
| 16 | (23S,25R)-25-hydroxyvitamin D3 26,23-peroxylactone | -0.01688 | 0.01673 | 0.983(0.952-1.016) | 0.317 | — |
| 17 | 23,24-didehydro-25-hydroxyvitamin D3 | -0.08845 | 0.09039 | 0.915(0.767-1.093) | 0.3318 | — |
| 18 | (22S)-1alpha,22,25-trihydroxy-26,27-dimethyl-23,23,24,24-tetradehydrovitamin D3 | -0.01746 | 0.01944 | 0.983(0.946-1.021) | 0.3729 | — |
| 19 | 1alpha,25-dihydroxy-19-norvitamin D3 | -0.06592 | 0.07363 | 0.936(0.81-1.082) | 0.3744 | — |
| 20 | 1alpha,25-dihydroxy-11alpha-methylvitamin D3 | -0.04538 | 0.05531 | 0.956(0.857-1.065) | 0.4153 | — |
| 21 | (23R,25R)-1alpha,25-dihydroxyvitamin D3 26,23-lactone | 0.02256 | 0.02935 | 1.023(0.966-1.083) | 0.4452 | — |
| 22 | 1alpha-hydroxy-23,24,25,26,27-pentanorvitamin D3 | -0.07345 | 0.0992 | 0.929(0.765-1.129) | 0.462 | — |
| 23 | 1alpha,25-dihydroxy-23-oxavitamin D3 | 0.03659 | 0.07049 | 1.037(0.903-1.191) | 0.6057 | — |
| 24 | 1alpha,25-dihydroxy-26,27-dimethyl-24a,24b-dihomovitamin D3 | 0.05059 | 0.11656 | 1.052(0.837-1.322) | 0.6659 | — |
| 25 | (1S)-1-hydroxy-23-oxo-24,25,26,27-tetranorcalciol | -0.01041 | 0.026 | 0.99(0.94-1.041) | 0.6902 | — |
| 26 | 16-Glutaryloxy-1alpha,25-dihydroxy-20-epivitamin D3 | 0.02777 | 0.07964 | 1.028(0.88-1.202) | 0.7286 | — |
| 27 | (23S)-23,25-dihydroxy-24-oxovitamin D3 | -0.01411 | 0.05618 | 0.986(0.883-1.101) | 0.8025 | — |
| 28 | 25-hydroxy-23-oxavitamin D3 | 0.03052 | 0.12297 | 1.031(0.81-1.312) | 0.8049 | — |
| 29 | 2alpha-Fluoro-19-nor-22-oxa-1alpha,25-dihydroxyvitamin D3 | -0.0059 | 0.0311 | 0.994(0.935-1.057) | 0.8503 | — |
| 30 | 1alpha,25-dihydroxy-9,11-didehydro-3-deoxyvitamin D3 | 0.02278 | 0.12208 | 1.023(0.805-1.3) | 0.8526 | — |
| 31 | (23S)-1alpha-hydroxy-25,27-didehydrovitamin D3 26,23-lactone | 0.00504 | 0.02726 | 1.005(0.953-1.06) | 0.8541 | — |
| 32 | 1alpha,25-dihydroxy-3-deoxy-19-nor-22-oxavitamin D3 | -0.0141 | 0.08974 | 0.986(0.827-1.176) | 0.8757 | — |
| 33 | 24,25-epoxy-1alpha-hydroxyvitamin D3 | 0.01092 | 0.07054 | 1.011(0.88-1.161) | 0.8775 | — |
| 34 | 24,24-Difluoro-1,25,26-trihydroxyvitamin D3 | 0.00662 | 0.04483 | 1.007(0.922-1.099) | 0.8832 | — |
| 35 | 1alpha,2alpha,25-trihydroxy-19-norvitamin D3 | -0.02685 | 0.23397 | 0.974(0.615-1.54) | 0.909 | — |
| 36 | 1alpha,2,25-trihydroxyvitamin D3 | -0.01301 | 0.13201 | 0.987(0.762-1.279) | 0.9218 | — |
| 37 | 1alpha,25-dihydroxy-26,27-dimethylvitamin D3 | -0.00773 | 0.10113 | 0.992(0.814-1.21) | 0.9393 | — |
| 38 | (23E)-1alpha,25-dihydroxy-16,17,23,24-tetradehydrovitamin D3 | 0.00273 | 0.04632 | 1.003(0.916-1.098) | 0.9532 | — |
| 39 | 1alpha-hydroxy-26,27-dimethylvitamin D3 | -0.00015 | 0.08963 | 1(0.839-1.192) | 0.9986 | — |
